# Operative Therapie bei perilunären Luxationen

**DOI:** 10.1007/s00113-025-01622-6

**Published:** 2025-08-26

**Authors:** A. Asmus, L. Harhaus-Wähner, F. Eichenauer

**Affiliations:** Klinik für Hand‑, Replantations- und Mikrochirurgie, BG-Klinikum Unfallkrankenhaus Berlin, Charité Universitätsmedizin Berlin, Warener Str. 7, 12683 Berlin, Deutschland

## Einleitung

Neben der raschen Beseitigung des Luxationszustandes ist eine zeitnahe operative Rekonstruktion bzw. Stabilisierung der komplexen Carpus-Verletzung erforderlich. Aufgrund der Variabilität und des teils erheblichen Ausmaßes der Schädigung sollte die Versorgung unter optimalen Bedingungen – sowohl in Bezug auf die Erfahrung des Operateurs als auch auf die technische Ausstattung – durchgeführt werden.

## Indikation

Die operative Behandlung einer perilunären Luxation oder Luxationsfraktur ist stets indiziert und stellt einen handchirurgischen Notfall dar. Neben der Reposition muss in dringlichen Fällen auch eine Entlastung des N. medianus erfolgen. Die definitive Versorgung kann dann auch zeitverzögert erfolgen. Bei Polytraumapatienten ist unbedingt eine interdisziplinäre Abstimmung erforderlich.

## Aufklärung

Die präoperative Aufklärung umfasst neben den allgemeinen Operationsrisiken insbesondere:

- Schädigung des N. medianus: Sowohl unfallbedingt als auch operativ kann es zu neurologischen Ausfällen kommen.
- Wundverschluss: Aufgrund der oft erheblichen Schwellung besteht das Risiko, dass ein primärer Wundverschluss nicht möglich ist. Es kann daher ein mehrzeitiges Verfahren mit temporärer Hautdeckung und späterem Wundverschluss erforderlich sein.
- Fixateur: Der Patient muss über die Möglichkeit einer vorübergehenden Behandlung mit einem Fixateur informiert werden.
- Mehrfache Eingriffe: Bei erweiterten ligamentären oder osteosynthetischen Versorgungen, verzögertem Weichteilverschluss und späteren Materialentfernungen kann mit mehreren Operationen gerechnet werden.
- Langfristige Risiken: Eine Schädigung des karpalen Gefüges infolge von Bandrupturen birgt ein erhöhtes Risiko für eine frühzeitige posttraumatische Arthrose, die im späteren Verlauf evtl. Teilarthrodesen oder weitere Eingriffe erforderlich macht. Auch das Auftreten von Pseudarthrosen sollte nicht unterschätzt werden. Dieses liegt je nach Literaturangebe zwischen 7 und 14 %. Dabei verbesserte sich das Outcome bei der Verwendung von Doppelgewindeschrauben gegenüber Drähten und der Versorgung von dorsal unter geringerer Beeinträchtigung der vaskulären Versorgung des Skaphoids. Bei lediglich geschlossen reponierten Verletzungen mit begleitenden Frakturen zeigt sich eine Pseudarthrosenrate von bis zu 50 % und sollte vermieden werden.

## Bildgebung

Zunächst erfolgt ein konventionelles Röntgen in 2 Ebenen (dorsopalmar und streng seitlich). Besonderer Fokus liegt dabei auf der seitlichen Darstellung, um die Karpalknochen in Bezug auf die Gelenkfläche des Radius und insbesondere die Position des Lunatums im Verhältnis zum Capitatum beurteilen zu können. Besteht eine Luxation, sollte – sofern möglich – eine sofortige Reposition angestrebt werden. Anschließend ist eine CT-Diagnostik empfehlenswert, um das genaue Ausmaß der Krafteinwirkung sowie evtl. Frakturen zu ermitteln.

Ein MRT ist nur indiziert, wenn – trotz hohem Verdacht auf das Verletzungsmuster – aktuell keine Luxation vorliegt (vgl. Abschnitt PLIND-Verletzung im CME-Beitrag „Perilunäre Luxationen“ von A. Asmus, L. Harhaus-Wähner und F. Eichenauer in dieser Ausgabe. 10.1007/s00113-025-01621-7); in diesem Fall kann die Untersuchung im kurzen Intervall erfolgen.

## Kurzkasuistik

**Abb. 1:**
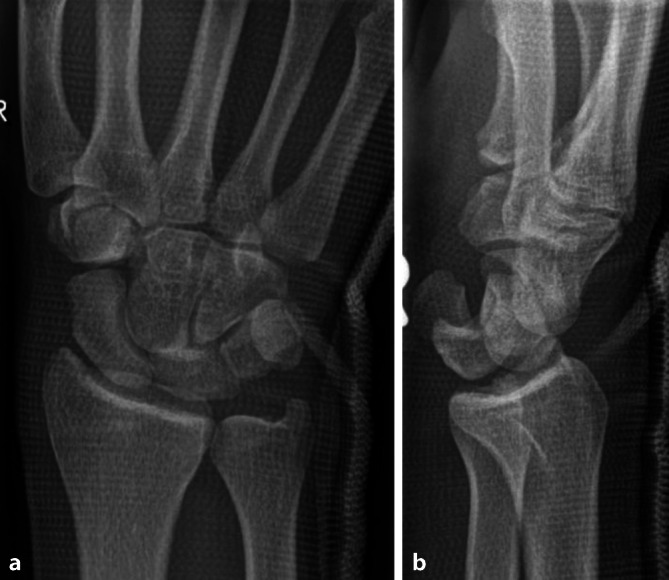
**a** p.a.-Ansicht: Gilula-Linien können im Mediokarpalgelenk nicht ohne Unterbrechung nachvollzogen werden als Hinweis auf eine Pathologie, **b** Im Seitbild erkennbare dorsale perilunäre „Lesser-arc“-Luxation, Stadium III nach Mayfield. Sichtbare Positionierung des Os capitatum dorsal des Os lunatum. Das Lunatum selbst steht noch im Radiokarpalgelenk

Ein 29-jähriger Motorradfahrer stürzt und wird mit dem Rettungswagen in die Notaufnahme gebracht. Die Sanitäter legen aufgrund starker Schmerzen im rechten Handgelenk bereits eine Schiene zur Ruhigstellung an. In der Notaufnahme zeigt sich eine ausgeprägte Schwellung um das Handgelenk; es besteht zwar keine Durchblutungsstörung, jedoch klagt der Patient über ein Kribbeln in den Fingern 1 bis 3. Aufgrund der erheblichen Schmerzen ist die Beweglichkeit des Handgelenks stark eingeschränkt, sodass umgehend eine Röntgenuntersuchung in 2 Ebenen durchgeführt wird (Abb. 1).

## Operationstechnik

### Reposition und Stabilisierung

#### Reposition in der Rettungsstelle

Analog zur Reposition einer distalen Radiusfraktur wird bei in 90° gebeugtem Ellenbogengelenk ein Zug von ca. 5–7 kg im Aushang appliziert. Nach ca. 10 min im Aushang reponiert sich die Fehlstellung meist selbst. Unterstützt werden kann die Reposition mit einem moderaten Druck von dorsal auf das Capitatum oder von palmar auf das Lunatum.

**Abb. 2:**
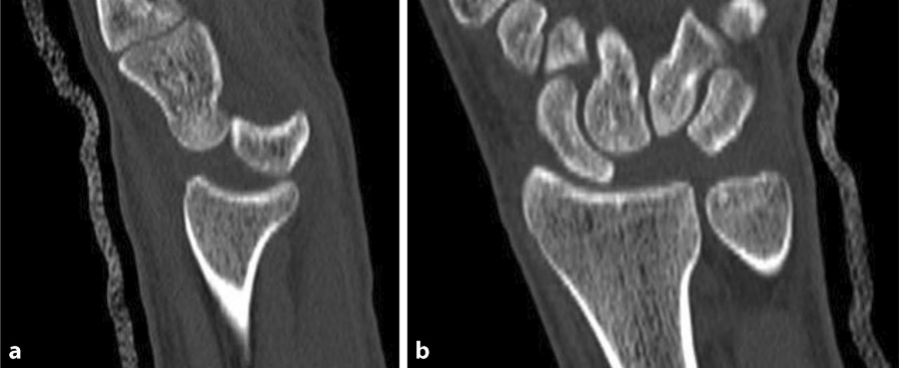
**a**, **b** Computertomographie vor Reposition zur weiteren Diagnostik, wobei weitere Frakturen ausgeschlossen wurden. **a** Sagittal mit fast aus dem Radiokarpalgelenk luxiertem Lunatum und **b** in koronarer Ebene mit Lücke in der proximalen Handwurzelreihe

Sind diese Maßnahmen aufgrund starker Schwellung, intensiver Schmerzen, Polytrauma oder mangelnder Kooperationsbereitschaft nicht durchführbar, sollte die Reposition im OP unter Vollnarkose erfolgen – in diesem Fall wird auch die CT als Teil der präoperativen Vorbereitung in der Luxationsstellung durchgeführt (Abb. 2).

#### Reposition in Vollnarkose

Entsprechend der Beschreibung nach Tavernier [1] wird bei der Reposition der Verletzungsmechanismus nachvollzogen. Zunächst erfolgt unter Extension und Zug das Lösen des Carpus aus der nach dorsal verhakter Stellung. Unter Flexion des Handgelenks und leichtem Druck von palmar auf das Lunatum kann der Carpus reponiert werden.

**Abb. 3:**
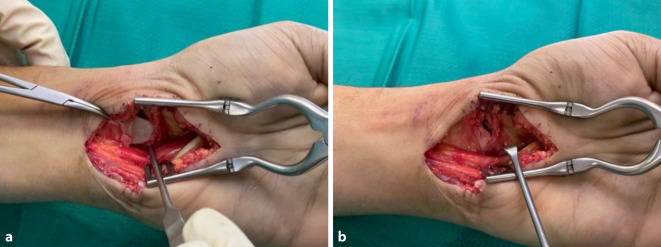
Karpaltunnelspaltung mit erweitertem Zugang. Beiseitehalten des N. medianus und der Beugesehnen. **a** Das noch vorluxierte Lunatum durch die zerrissenen palmaren Bänder der Handwurzel. **b** Reposition des Lunatum durch die Handgelenkkapsel und extrinsischen Ligamente

Ist das Lunatum nach palmar aus dem Handgelenk herausluxiert, führen geschlossene Repositionsmanöver nicht zum Erfolg. In diesem Fall und bei klinisch nachgewiesener Druckschädigung des N. medianus ist eine Entlastung mittels Karpaltunnelspaltung angezeigt. Dabei kann im Rahmen der Eröffnung des Karpaltunnels ein nach palmar in den Karpalkanal luxiertes Lunatum dargestellt und schonend durch die Luxationslücke des palmaren Bandapparats reponiert werden (Abb. 3). Nach erfolgreicher Reposition des Os lunatum erfolgt der Verschluss der Luxationslücke im palmaren Bandapparat mittels resorbierbarer Naht.

Nach erfolgreicher Reposition und initialem Verschluss der Gelenkkapsel ist – abhängig von der intraoperativen Stabilität – zu entscheiden, ob eine primäre definitive Versorgung oder ein zeitverzögerter, sekundärer Eingriff sinnvoller ist. Faktoren wie das Verletzungsausmaß (assoziierte Frakturen, Bandzerstörung), Begleitverletzungen (z. B. beim Polytrauma) und die Erfahrung des Operateurs spielen hierbei eine wesentliche Rolle. Wird die weitere Versorgung zweizeitig durchgeführt, erfolgt zur Stabilisierung entweder die Anlage eines Fixateur externe oder bei geringer Redislokationstendenz die Anlage eines zirkulär gespaltenen Unterarmgipses.

Diese oben genannten erforderlichen Erstmaßnahmen der geschlossenen Reposition und ggf. Karpaltunnelspaltung können auch in einem Nichthandtraumazentrum durchgeführt werden.

### Zugang

**Abb. 4:**
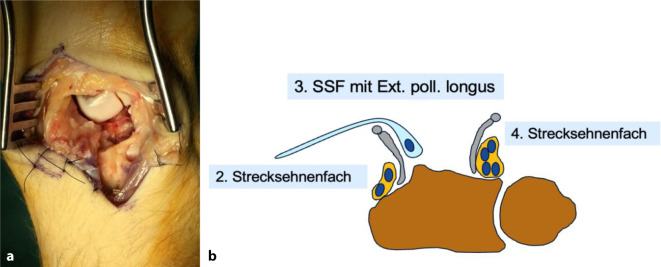
**a** Zugang von dorsal mit sichtbarer Lücke proximal des Capitatums bei noch nicht reponiertem Lunatum, **b** schematischer Zugang zum Handgelenk von dorsal (modifiziert nach [2])

Die Reposition und Stabilisierung erfolgen meist über einen dorsalen Zugang zum Handgelenk unter Eröffnung des 4. Strecksehnenfachs und der Gelenkkapsel (Abb. 4). Optional kann hierbei am Boden des 4. Strecksehnenfachs eine Denervierung des N. interosseus posterior (NIP) zur Schmerzreduktion durchgeführt werden. Die sog. selektive Denervierung des NIP ist allgemein aufgrund der propriozeptiven Funktion des Nervs in Bezug auf den SL-Bandkomplex umstritten, jedoch ist dieser bei den meisten perilunären Luxationen ohnehin hochgradig beschädigt bzw. zerstört.

### Osteosynthese und Transfixierung

**Abb. 5:**
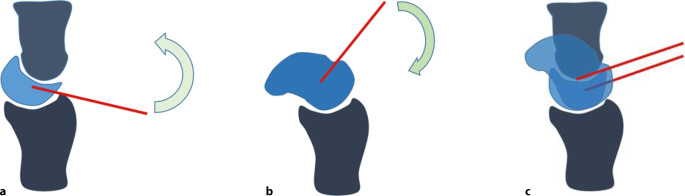
Schematische Darstellung der Reposition von Lunatum und Skaphoideum nach Ruptur des SL-Band-Komplexes. Das Lunatum steht nach dorsal und das Skaphoid nach palmar flektiert

- Frakturfixierung: Abhängig vom Verletzungsausmaß werden Frakturen der Carpalia meist über Doppelgewindeschrauben und/oder Bohrdrähte stabilisiert.
- Transfixierung: Anschließend erfolgt die Transfixierung der Handwurzelknochen. Hierzu werden Drähte (1,2 mm oder 1,4 mm) als Repositionshilfen („Joysticks“) in das Os lunatum und das Os scaphoideum eingebracht. Der Skaphoiddraht sollte schräg von distal nach proximal, der Lunatumdraht von proximal nach distal platziert werden (Abb. 5).

Bei frischen Verletzungen kann man sich beim offenen Repositionsmanöver an den erkennbaren Bandresten orientieren.

**Abb. 6:**
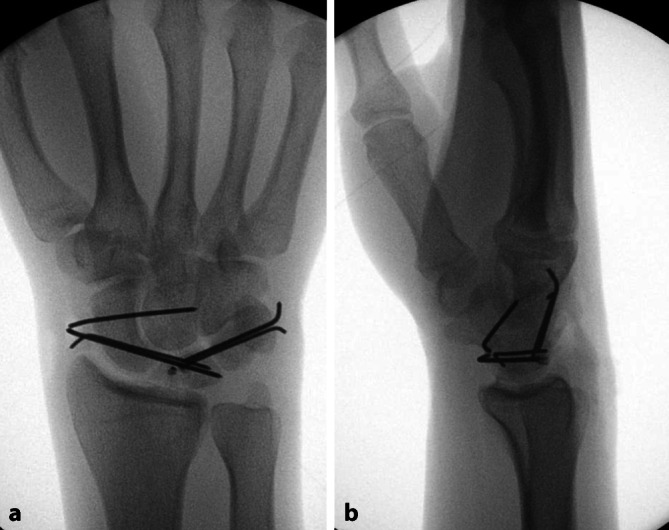
**a**,**b** Nach Aufrichtung dann Fixierung zwischen Skaphoid/Lunatum und Lunatum/Triquetrum mit je 2 Drähten sowie einem Draht zwischen Skaphoid und Capitatum, einschließlich Knochenanker für die Adaptation der Bandreste

Das reponierte skapholunäre Gelenk wird mittels ein oder zwei Bohrdrähten transfixiert. Je nach Luxationslinie können zusätzliche Stabilisierungspunkte zwischen Os triquetrum und Os lunatum bzw. zwischen Os scaphoideum und Os capitatum erforderlich sein (Abb. 6). Sämtliche rekonstruierbaren Bandanteile werden im Rahmen des offenen Verfahrens durch Nahtadaptation, Knochenanker oder transossäre Refixierung gesichert.

### Wundverschluss und Abschluss

Der primäre Wundverschluss sollte angestrebt, jedoch nicht erzwungen werden. Bei ausgeprägter Schwellung ist ein zweizeitiger Wundverschluss mit temporärer Abdeckung (z. B. mittels Hautersatzmaterial) oft sinnvoller. Abschließend wird – je nach Stabilität – entweder eine Gipslonguette oder ein Fixateur externe angelegt.

### Minimal-invasive Alternativen

**Abb. 7:**
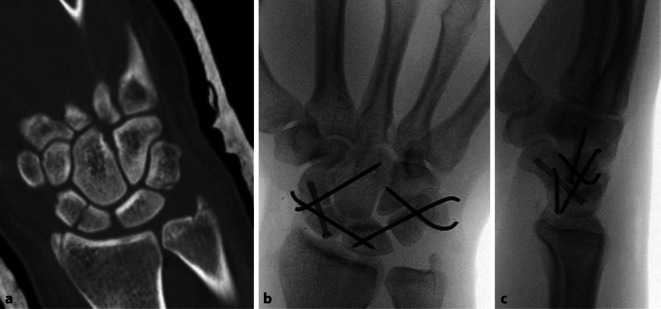
**a**–**c** Versorgung einer begleitenden Skaphoidfraktur von dorsal bei einer „Greater-arc“-Verletzung

Alternativ zum offenen dorsalen Zugang ist nach erfolgter Reposition (ggf. palmar offen mit Spaltung des Karpaltunnels) auch eine minimal-invasive, teils arthroskopisch assistierte Transfixation und Osteosynthese möglich. Hierbei wird in der Regel auf eine direkte Refixierung der Bandstümpfe verzichtet, sofern eine korrekte Reposition und perkutane Transfixierung der Gelenke gewährleistet sind (Abb. 7).

Die Vorteile liegen in der Schonung der während der Luxation intakt verbliebenen palmaren und dorsalen Gelenkkapsel und Bandstrukturen. Allerdings ist eine geschlossene Versorgung kontraindiziert bei grob dislozierten, nicht ausreichend reponiblen knöchernen Bandausrissen, vornehmlich auf der Streckseite.

## Operationsprinzipien

Das Ziel besteht in einer möglichst anatomischen Reposition, die offen, minimal-invasiv oder arthroskopisch assistiert erfolgen kann. Die Maßnahmen umfassen:

- stabile osteosynthetische Versorgung von Frakturen,
- Transfixierung des Carpus,
- Versorgung von Bandrupturen.

In bestimmten Fällen – bedingt durch den Patientenzustand oder bessere Versorgungsmöglichkeiten – kann die definitive operative Behandlung nach einer schnellen geschlossenen Reposition um einige Stunden oder Tage verzögert werden.

## Fehlerquellen

### Diagnostisches Versäumnis

Häufig wird die perilunäre Luxationsverletzung übersehen, was zu einer verzögerten operativen Versorgung führt. Ein Übersehen der Verletzung wird in der Literatur zwischen 16 % und 25 % angegeben, wobei sich die Rate in den letzten Jahren durch vermehrte Vigilanz und bessere Diagnostik zunehmend verbessert. Dies ist oft auf die Seltenheit der Verletzung und mangelnde Erfahrung im Erkennen typischer radiologischer Zeichen zurückzuführen.

### Operative Fehler

Fehler können in einer unzureichenden Reposition und Fixation der Carpalia mittels Bohrdrähten oder osteosynthetischen Verfahren liegen. Die Transfixierung muss sämtliche Komponenten der Instabilität berücksichtigen. Bei präoperativ unklaren Fällen ist eine dynamische Untersuchung in Narkose im OP zu empfehlen. In maximaler Ulnarduktion zeigt sich Aufweitung des Spalts zwischen Skaphoid und Lunatum und ermöglicht die Beurteilung des SL-Band-Komplexes. Eine Instabilität des LT-Band-Komplexes stellt sich durch eine vermehrte Translation des Triquetrums dar.

### Postoperative Kontrolle

Zur Überprüfung des Operationsergebnisses empfiehlt sich ein Kontroll-CT, da fehlerhaft platzierte Drähte oder grobe Fehlstellungen häufig korrigiert werden müssen. Eine definitive Versorgung sollte stets von einem erfahrenen Operateur durchgeführt oder der Patient in eine spezialisierte handchirurgische Klinik verlegt werden.

## Komplikationen

### Frühkomplikationen

- Akutes Karpaltunnelsyndrom und/oder Kompartmentsyndrom
- Irritationen des R. superficialis n. radialis sowie des R. dorsalis n. ulnaris durch das perkutane Einbringen der Drähte, die zu einem chronischen Schmerzsyndrom führen können. Günstiger ist es, den Hautschnitt etwas größer anzulegen, die Nervenäste darzustellen und die Drähte sicher zu platzieren.

### Langzeitkomplikationen

- Anhaltende karpale Instabilität
- Pseudarthrosen
- Frühzeitige posttraumatische Arthrose
- Posttraumatische Lunatumnekrose (selten, ca. 1 % der Fälle, vorwiegend nach vollständiger palmarer Luxation im Stadium IV/3)

## Nachbehandlung

Die Ruhigstellung erfolgt in der Regel 8 Wochen lang mittels Gips/Orthese (bei Einschluss des ersten Mittelhandknochens) oder Fixateur externe. Anschließend werden die zur temporären Transfixierung verwendeten Bohrdrähte entfernt, und es beginnt eine Physio- bzw. Ergotherapie. Eine sukzessive Steigerung der Belastung sollte frühestens 12 Wochen postoperativ erfolgen.
